# *Qiliqiangxin* Improves Cardiac Function through Regulating Energy Metabolism via HIF-1*α*-Dependent and Independent Mechanisms in Heart Failure Rats after Acute Myocardial Infarction

**DOI:** 10.1155/2020/1276195

**Published:** 2020-06-12

**Authors:** Yanyan Wang, Mingqiang Fu, Jingfeng Wang, Jingjing Zhang, Xueting Han, Yu Song, Yuyuan Fan, Kai Hu, Jingmin Zhou, Junbo Ge

**Affiliations:** ^1^Department of Cardiology, Shanghai Institute of Cardiovascular Diseases, Zhongshan Hospital, Fudan University, Shanghai, China; ^2^Department of Cardiology, Zoucheng Hospital, Affiliated Hospital of Jining Medical University, Jining, Shandong, China; ^3^North Sichuan Medical College, Nanchong, Sichuan, China

## Abstract

The present study is aimed at investigating whether *Qiliqiangxin* (QL) could regulate myocardial energy metabolism in heart failure rats after acute myocardial infarction (AMI) and further exploring the underlying mechanisms. AMI was established by ligating the left anterior descending coronary artery in adult male SD rats. AMI rats with ejection fraction (EF) < 50% at two weeks after the operation were chosen as heart failure rats for the main study. Rats were randomized into the sham, MI, MI+QL, and MI+QL+2-MeOE2 groups. The results showed that compared with the MI group, QL significantly improved cardiac function, reduced serum NT-proBNP level, and alleviated myocardial fibrosis. QL also increased myocardial capillary density by upregulated protein expressions of vascular endothelial growth factor (VEGF) and CD31 by regulating the HIF-1*α*/VEGF pathway. Moreover, QL promoted ATP production, glucose uptake, and glycolysis by upregulating HIF-1*α* and a series of glycolysis-relevant enzymes in a HIF-1*α*-dependent manner. QL also improved myocardial glucose oxidation enzyme expression and free fatty acid uptake by a HIF-1*α*-independent pathway. Our results indicate that QL treatment improves cardiac function through regulating glucose uptake, FFA uptake, and key enzymes of energy metabolism via HIF-1*α*-dependent and independent mechanisms.

## 1. Introduction

Heart failure (HF) is one of the most important cardiovascular diseases (CVD) that is associated with high morbidity and mortality [1]. Myocardial ischemia is one of the major causes of heart failure. Although reperfusion therapy has improved the prognosis of ST-elevation myocardial infarction (STEMI), the progression to HF can still occur in 30% of patients post myocardial infarction (MI) [1]. Despite recent advances in medical and surgical therapy, patients with HF have a 5-year mortality rate of around 50% [2, 3]. Thus, new approaches for HF treatments are needed. Targeting myocardial metabolism might be an attractive therapeutic option for HF. There is a vicious metabolic circle in heart failure as the activated adrenergic system could lead to an increase in the serum free fatty acid (FFA), which inhibits mitochondrial function at the level of acyl carnitine transferase (ACT), thus inhibiting fatty acid (FA) oxidation and synthesis of ATP [4, 5]. Furthermore, this increase in the serum FFA also inhibits pyruvate dehydrogenase (PDH) activity via the Randle cycle to promote anaerobic glycolysis rather than oxidative metabolism [6]. Previous studies have shown that the metabolic shift from oxidative metabolism (including FA and glucose oxidative metabolism) to glycolysis and the substrate shift from primarily fatty acids to glucose could result in decreased myocardial ATP production, which might play a crucial role in the development of heart failure [7, 8]. Accordingly, there is accumulating evidence that modulating cardiac energy metabolism, either by increasing glucose oxidation or by decreasing serum FFA concentrations and FA oxidation, can improve cardiac function [9, 10].

Hypoxia-inducible factor 1*α* (HIF-1*α*) is an important cellular adaptation mediator against hypoxia, which can regulate the delivery of oxygen by promoting vascular endothelial growth factor (VEGF) expression and angiogenesis [11]. Furthermore, HIF-1*α* can also modulate energy metabolism by activating the expression of glucose metabolic key enzymes (e.g., hexokinase 2 (HK2), pyruvate dehydrogenase K1 (PDK1), lactic dehydrogenase (LDHA)), and glucose transporter vectors 1/4 (GLUT1/4)) and mitochondrial proteins [12]. Recent studies have suggested that upregulated HIF-1*α* activity is closely linked with cardioprotective effects induced by myocardial infarction; the related mechanism promotes angiogenesis [13] and improves metabolism [14]. Another study also showed that the HIF-1*α* activity in endothelial cells was required for the cardioprotective effects induced by ischemic preconditioning (IPC), while in HIF-1*α*-/- mice, the cardioprotective effect of IPC was diminished [15]. This evidence suggested that the upregulation of HIF-1*α* might be a potential therapeutic option for treating heart failure post AMI.

*Qiliqiangxin* is a compound preparation of traditional Chinese medicine, which is extracted from 11 types of Chinese herbs, including Radix Astragali, Aconite Root, Ginseng, Salvia miltiorrhiza, Semen Lepidii Apetali, Cortex Periplocae Sepii Radicis, Rhizoma Alismatis, Carthamus tinctorius, Polygonatum Odorati, Seasoned Orange Peel, and Rumulus Ginnamomi. The 17 chemical constituents of QL mainly include ginsenoside Rg1, phenolic acids, flavonoids and alkaloids, astragaloside, calycosin-7-glucoside, and sinapine [16]. QL has shown satisfactory efficacy in the treatment of HF patients in China [17]. Previous studies found that QL improved cardiac metabolism and mitochondrial function in heart failure induced by pressure overload, while QL also enhanced the metabolism of H9C2 cardiomyocytes by regulating peroxisome proliferator-activated receptor gamma (PPAR*γ*) coactivator-1*α* (PGC-1*α*) expression [18, 19]. Our previous studies also demonstrated that QL could increase cardiac angiogenesis both in rats post MI and in hypoxic cardiac microvascular endothelial cells [16, 20]. However, it remains unknown to what extent the changes in energy metabolism contributed to the beneficial effects of QL in MI rats.

The present study is aimed at investigating the effects of QL on energy metabolism in rats with experimental MI and exploring whether HIF-1*α* signaling is actively involved in these effects.

## 2. Materials and Methods

### 2.1. Experimental Animals

Adult male SD rats (with an average weight of 200 ± 20 g) were purchased from the Shanghai SLAC Laboratory Animal Co., Ltd. (Shanghai, China). Rats were housed in a temperature-controlled laboratory animal center (22–24°C) under circadian conditions with free access to standard chow and tap water. The study was approved by the ethical committees of the Zhongshan Hospital Affiliated to Fudan University, and all the experimental procedures were performed in accordance with the Guide for the Care and Use of Laboratory Animals published by the US National Institutes of Health (NIH Publication No. 85-23, revised 1996).

### 2.2. Induction of Myocardial Infarction in Rats

After 1 week of adaptive feeding, the rats were anesthetized with sodium pentobarbital (30 mg/kg, intraperitoneally), intubated, and connected to a SAR-830/A Small Animal Ventilator (CWE, Inc., Weston, WI, USA). Thereafter, MI was created by exteriorizing the heart through a left thoracic incision, placing a 6-0 silk 3–4 mm below the tip of the left auricle, and making a slipknot. The complete occlusion of the vessel was confirmed by the presence of myocardial blanching in the perfusion bed. After ligation, the chest of the animal was closed with three 5-0 prolene sutures, which was followed by 4-0 polyester sutures to close the skin. For the sham group, the needle was passed around the artery without ligation.

Two weeks later, the rats that survived after the MI operation (the survival rate was 65%) were subjected to an echocardiographic examination and those with left ventricular ejection fraction (EF) of <50% were chosen for subsequent studies. The distribution of EF in MI rats before treatment was 35%-45%, and there was no significant difference between groups; those rats whose EF was not at this range were excluded.

### 2.3. Experimental Groups

Rats were randomly divided into the following groups (*n* = 7 per group). The sham group and the MI group both received the same volume of 0.5% sodium carboxymethyl cellulose (CMC-Na). The other two groups were the MI+QL group and the MI+QL+2-MeOE2 group. QL at 0.5 g/kg was given daily by gavage, while 2-MeOE2 (HIF-1*α* inhibitor) at 30 mg/kg was given daily by gavage. According to our previous study [16], we planned to give this medicine for 6 weeks.

The QL compound was supplied by Yiling Pharmaceutical Corporation (Shijiazhuang, China); the compound was suspended in 0.5% sodium carboxymethyl cellulose (CMC-Na) at a 10% concentration before administration.

### 2.4. Small-Animal Positron Emission Tomography Imaging Analysis

Micro-PET/CT (Metis PET, Madic, Shandong, China) was performed before and 6 weeks after various interventions to evaluate the changes in the myocardial glucose metabolism. After anesthesia with sodium pentobarbital (30 mg/kg, IP), 2-deoxy-2-fluoro-D-glucose (^18^F-FDG) (approximately 1250 *μ*Ci/kg) was injected via the tail vein of the rats. One hour later, the myocardial glucose metabolism level was examined. The emission scan was acquired in one bed position with a total acquisition time of 30 minutes. During the entire PET examination process, anesthesia was maintained with 1.5% isoflurane delivered in pure oxygen at a rate of 1.2 l/min via a face mask and the rats were placed within the aperture of the tomography in a prone position. The Metis Viewer Version 1.0 for Windows was used for image analysis. After this, the standardized uptake value (SUV), which is equal to the PET count/injected radioactivity per body weight (Bq/g), was calculated and normalized according to body weight and the injected dose of ^18^F-FDG. Finally, the mean standardized ^18^F-FDG-uptake values (SUV_mean_) were obtained by a professional observer.

### 2.5. Echocardiography and Blood and Myocardial Sample Collection

Echocardiography was performed under anesthesia 6 weeks after the interventions. The rats were placed in the proper posture after the precordial region was shaved. M-mode images of LV long axis were obtained at the level of the papillary muscle tips using an animal specific instrument (VisualSonics Vevo770; VisualSonics Inc., Toronto, ON, Canada) as previously described [21–23]. Left ventricular internal diameter at end diastole (LVIDd) and left ventricular internal diameter at end systole (LVIDs), fractional shortening (FS), and EF were measured. Parameters were obtained and averaged from three consecutive cardiac cycles and performed by one experienced echocardiographer who was blinded to the treatment protocol.

Blood samples were collected by cardiac puncture and centrifuged at 3000 rpm/min for 20 minutes at 4°C. After this, the serum was collected and stored at -80°C. The heart was immediately excised after euthanasia, which was irrigated clean with cold saline. The ventricular transection was separated into 2 equal parts. The top half of the transversely dissected heart tissue was fixed in 4% paraformaldehyde, while the other half of the tissue was divided into the infarct border zone and noninfarcted left ventricular myocardium, which was stored at -80°C. After 6 hours, the tissues fixed in 4% paraformaldehyde were divided into 2 equal parts again: the top part was made into frozen embedded tissues immediately, while the other part was fixed for 48 hours and made into paraffin-embedded tissues used for hematoxylin/eosin (H&E) and Sirius red staining.

### 2.6. Serum Concentrations of NT-proBNP and Free Fatty Acid (FFA) Level

The serum NT-proBNP level was measured using the enzyme-linked immunosorbent assay (ELISA) according to the manufacturer's instructions (Cloud-Clone Corp., USA). The serum FFA level was measured by a commercial assay kit according to the manufacturer's instructions (BioSino Bio-Technology & Science Inc., Beijing, China).

### 2.7. Detection of ATP Production and Citrate Synthase (CS) Activity

The ATP level was measured using an ATPlite assay kit (Beyotime Biotechnology, Shanghai, China). The tissues around the border zone (20 mg) were homogenized in 200 *μ*l of the ATP assay buffer before being centrifuged at 12,000 g for 5 min at 4°C. A total of 100 *μ*l of samples or standards was mixed with 100 *μ*l of the ATP Reaction Mix in duplicate wells of a 96-well plate. Luminescence was detected using a fluorescent plate reader (Synergy™ H4, BioTek Instruments, Inc., USA).

In the present study, we detected the CS activity to evaluate the effect of QL on glucose oxidative metabolism according to the manufacturer's instructions (Sigma-Aldrich Co. LLc., USA). Briefly, the border zone tissue samples (10 mg) were homogenized in 100 *μ*l of the CS Assay Buffer, kept on ice for 10 minutes, and centrifuged at 10,000 g for 5 minutes at 4°C. We added 50 *μ*l of the samples (containing 20 *μ*l of supernatants and 30 *μ*l of CS Assay Buffer) or 50 *μ*l of the standard buffer to the 96-well plate. After this, we added 50 *μ*l of the reaction mix, which includes the CS Assay Buffer, CS Developer, and CS Substrate Mix, to each well. We incubated the plate at 25°C, before both the initial and final absorbance at 412 nm was measured. Finally, we calculated the CS activity according to the following mathematical formula: CS activity (nmol/min/*μ*l) = Sa/(reaction time) × Sv, where Sa represents the amount of GSH (nmol) generated in the sample wells between Tinitial and Tfinal from the standard curve and Sv is the sample volume. Finally, we used the following formula: reactive time = Tfinal − Tinitial.

### 2.8. Histology and Fibrosis Assessment

After 6 weeks of the intervention, hearts were excised, cut into 2 mm thick transverse sections, and fixed in 4% phosphate-buffered formalin. After this, the paraffin-embedded tissues were cut into 5 *μ*m slices and stained with hematoxylin/eosin (H&E) and Sirius red. Sections were examined by light microscopy, and the images were analyzed with ImageJ software to quantify the collagen percentage as previously described [24]. Images of five randomly chosen fields round the border zone were photographed under a microscope (Leica, Germany). The myocardial collagen volume fraction (CVF) was obtained by calculating the ratio of fibrotic area (red) to the total myocardial area using ImageJ software (NIH, Bethesda, MD, USA).

### 2.9. Detection of Myocardial Microvessel Density by Immunofluorescence Staining

CD31 was used to evaluate the level of angiogenesis. The frozen embedded tissues were cut into 8 *μ*m frozen slices. Frozen tissue sections were placed at room temperature for 30 minutes and washed with phosphate-buffered saline (PBS) three times before immunofluorescence staining. After blocking with goat serum for 30 min, the sections were incubated with rabbit anti-rat CD31 (1 : 20 dilution, Santa Cruz Biotechnology, Inc., USA) primary antibody overnight at 4°C. On the next day, after being washed with PBS four times (10 min for each), the slides were incubated with goat anti-rabbit secondary antibodies (Alexa Fluor 555, Invitrogen, Carlsbad, CA, USA) for 1.5 hour at 37°C in the dark. Nuclei were counterstained with DAPI. Images of five randomly chosen fields round the border zone were photographed under a microscope (Leica, Germany), and the vessel density was analyzed by ImageJ software (NIH, Bethesda, MD, USA).

### 2.10. Fluorescent Quantitative RT-PCR Analysis

The total RNA was extracted from 10 mg of the left ventricular infarction border zone tissue using the TRIzol Reagent according to the manufacturer's protocol (Invitrogen, Carlsbad, CA, USA). The total RNA (1 *μ*g) was reverse transcribed into cDNA using a RT Reagent Kit (TaKaRa, Tokyo, Japan). Real-time PCR was performed with the SYBR® Premix Ex Taq™ kit in a 10 *μ*l reaction volume (TaKaRa, Tokyo, Japan). The primers used for PCR are shown in Table 1. qRT-PCR was performed on the CFX Connect ™ Real-Time System (Bio-Rad Laboratories, Inc., California, USA). The standard 2^−ΔΔCt^ relative quantification method was applied with *β*-actin used as the endogenous control.

### 2.11. Western Blot Analysis

Approximately 10 mg of the left ventricular border zone tissue samples was lysed and homogenized in 200 *μ*l of the lysis buffer on ice, which was followed by centrifugation at 12,000 g for 20 min at 4°C. Protein concentration was measured by the BCA protein assay (Beyotime Biotechnology, Shanghai, China). Aliquots of 30 *μ*g of the protein lysates were electrophoresed on 8%, 10%, or 12% SDS-PAGE gels and then transferred to PVDF membranes (Millipore, Billerica, MA, USA). After being blocked with 5% bovine serum albumin (BSA) for 1 hour at room temperature, the blots were incubated overnight at 4°C with specific rabbit/mouse anti-rat antibodies for HIF-1*α* (Novus International Inc., USA, 1 : 1000), GLUT4 (Cell Signaling Technology, USA, 1 : 800), PKM2 (Cell Signaling Technology, USA, 1 : 1000), LDHA (Cell Signaling Technology, USA, 1 : 1000), PDH (Cell Signaling Technology, USA, 1 : 1000), CS (Cell Signaling Technology, USA, 1 : 1000), CD36 (Cell Signaling Technology, USA, 1 : 1000), PGC-1*α* (Cell Signaling Technology, USA, 1 : 1000), VEGF (Cell Signaling Technology, USA, 1 : 1000), VEGFR2 (Cell Signaling Technology, USA, 1 : 1000), and CD31 (Santa Cruz Biotechnology, Inc., USA, 1 : 200). On the next day, membranes were washed with TBST three times (10 min for each) before being incubated with horseradish peroxidase- (HRP-) conjugated secondary antibodies (1 : 10000, Kangchen, China) for 2 hours at room temperature. The HRP-conjugated monoclonal rabbit anti-rat *β*-actin antibody (1 : 10000, Kangchen, China) was used to detect *β*-actin levels. Finally, the antibody complexes were visualized and quantified by the Gel Doc™ XR+ System (Bio-Rad Laboratories, Inc., California, USA) and Image Lab Software. The results were expressed as density values that were normalized to *β*-actin.

### 2.12. Statistical Analyses

Data were presented as mean ± SEM. For the comparison between two groups, the differences in the mean values were evaluated by Student's *t*-test and the Mann-Whitney *U* test. For the comparison among three or more groups, differences were determined by one-way ANOVA with Tukey's post hoc test. A value of *p* < 0.05 was considered to be statistically significant. All statistical analyses were performed with SPSS 17.0 software.

## 3. Results

### 3.1. QL Treatment Preserved Cardiac Function and Decreased the Level of Serum NT-proBNP in MI Rats in a HIF-1*α*-Dependent Manner

**Figure 1:**
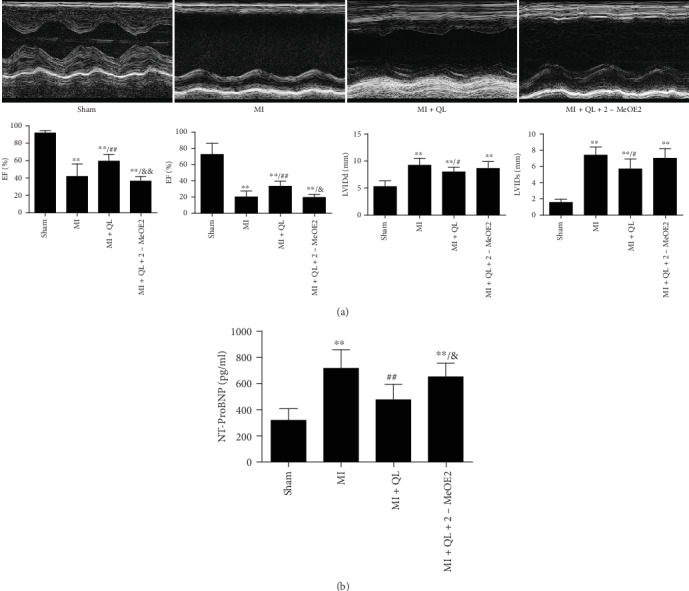
Qiliqiangxin (QL) improved cardiac function and decreased serum NT-proBNP levels via the HIF-1*α* signaling pathway. (a) QL improved cardiac function with preserved left ventricular ejection fraction (EF), left ventricular fractional shortening (FS), left ventricular internal diameter at end-diastole (LVIDd), and left ventricular internal diameter at end systole (LVIDs), while the HIF-1*α* inhibitor partly suppressed these protective effects of QL (*n* = 7/group). (b) QL decreased serum NT-proBNP levels in a HIF-1*α*-dependent manner (*n* = 7/group). Data are expressed as means ± SEM. ^∗^*p* < 0.05 and ^∗∗^*p* < 0.01 compared with the sham group; ^#^*p* < 0.05 and ^##^*p* < 0.01 compared with the MI group; ^&^*p* < 0.05 and ^&&^*p* < 0.01 compared with the MI+QL group.

Compared with the sham group, increased LVIDd, LVIDs, and NT-proBNP levels, in addition to decreased EF and FS were found in the MI group. QL treatment for 6 weeks significantly improved cardiac function and decreased NT-proBNP levels, while these protective effects were partly attenuated in the presence of a HIF-1*α* inhibitor (Figures 1(a) and 1(b)).

### 3.2. QL Ameliorated Myocardial Derangement and Fibrosis via a HIF-1*α*-Dependent Pathway

**Figure 2:**
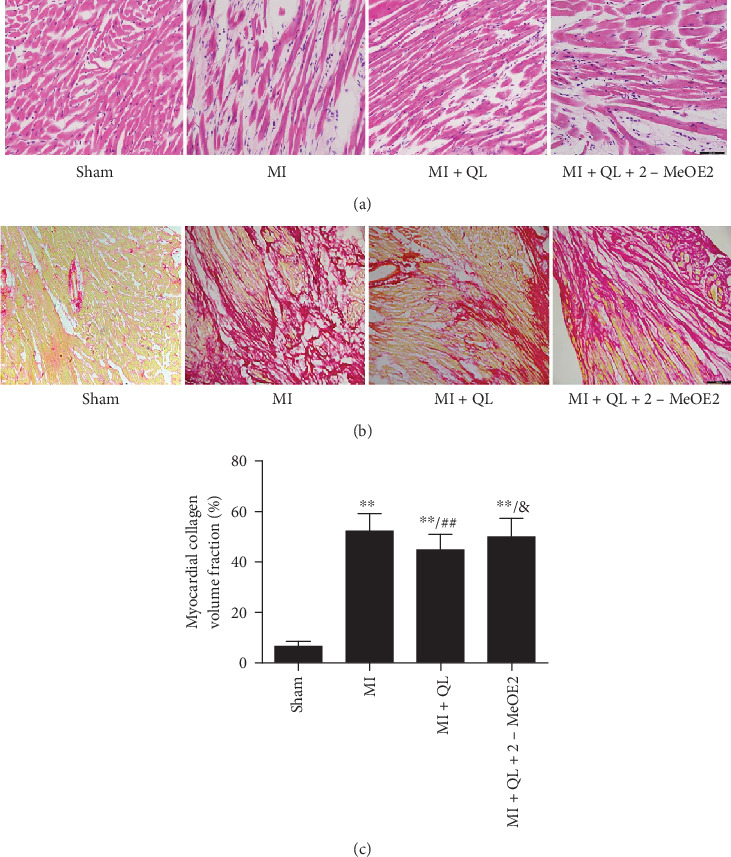
QL preserved myocardial structure and inhibited cardiac fibrosis via the HIF-1*α*-dependent pathway. (a) QL preserved the arrangement of myocardial fibers, which were obviously irregularly arranged in the MI group (20x, scale bar = 100 *μ*m, *n* = 4/group). (b and c) QL attenuates cardiac fibrosis with decreased cardiac fibrosis compared with the MI group (10x, scale bar = 200 *μ*m, *n* = 4/group). Data are expressed as means ± SEM. ^∗^*p* < 0.05 and ^∗∗^*p* < 0.01 compared with the sham group; ^#^*p* < 0.05 and ^##^*p* < 0.01 compared with the MI group; ^&^*p* < 0.05 and ^&&^*p* < 0.01 compared with the MI+QL group.

As shown in Figure 2(a), the surviving myocardial cells in the infarct border zone were obviously irregularly arranged, the myocardial cells displayed lysis and breakage of cardiac muscle fibers, while numerous infiltrated inflammatory cells were detectable, which was partially reversed by QL treatment. However, cotreatment with the HIF-1*α* inhibitor 2-MeOE2 attenuated the beneficial effects of QL again. Myocardial fibrosis is a hallmark of cardiac remodeling after AMI. Sirius red staining revealed significantly enhanced cardiac fibrosis in the MI group compared to the sham group, while QL significantly alleviated cardiac fibrosis, and this effect could be partially blocked by treatment with 2-MeOE2 (Figures 2(b) and 2(c)).

### 3.3. QL Treatment Promoted Myocardial Glucose Uptake and ATP Production via a HIF-1*α* Signaling Pathway

**Figure 3:**
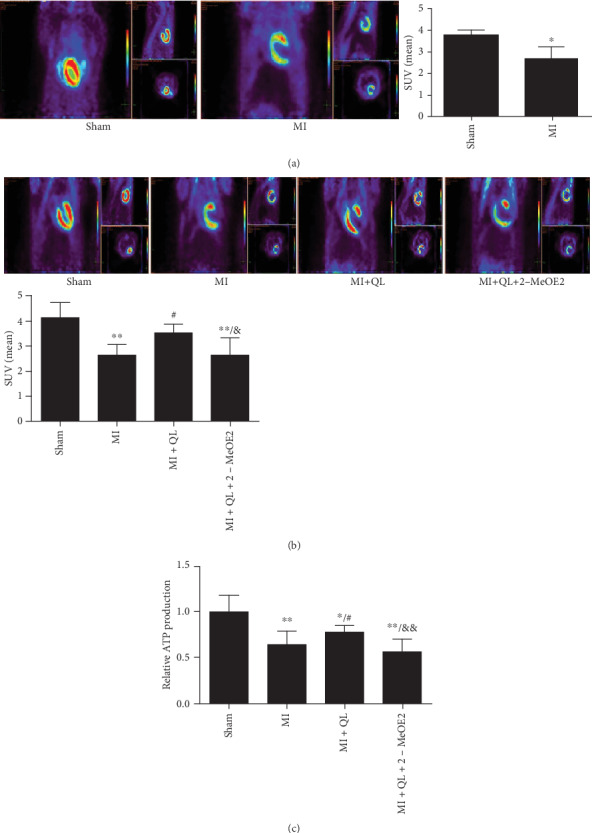
QL promoted myocardial glucose uptake and ATP production via the HIF-1*α* signaling pathway. (a) Animals of the MI group showed a significantly lower myocardial ^18^F-FDG uptake (exhibited as SUV mean) than those of the sham group before drug intervention. After 6 weeks of treatment, the myocardial ^18^F-FDG uptake (SUV mean) (b) and ATP production (c) were significantly increased in the MI+QL group compared with the MI group, while the HIF-1*α* inhibitor attenuated these effects (*n* = 7/group). Data are expressed as means ± SEM. ^∗^*p* < 0.05 and ^∗∗^*p* < 0.01 compared with the sham group; ^#^*p* < 0.05 and ^##^*p* < 0.01 compared with the MI group; ^&^*p* < 0.05 and ^&&^*p* < 0.01 compared with the MI+QL group.

Before drug administration, we used PET to detect the cardiac glucose uptake of MI rats and sham rats. The results showed that compared with the sham group, the myocardial glucose uptake was significantly lower in MI rats (Figure 3(a)).

After 6 weeks, the results revealed significantly higher myocardial ^18^F-FDG uptake and ATP production in the MI+QL group compared with the MI group, while treatment of the specific HIF-1*α* inhibitor significantly blocked these effects. This suggests that the HIF-1*α* pathway played a critical role in QL-induced metabolic improvement (Figures 3(b) and 3(c)).

**Figure 4:**
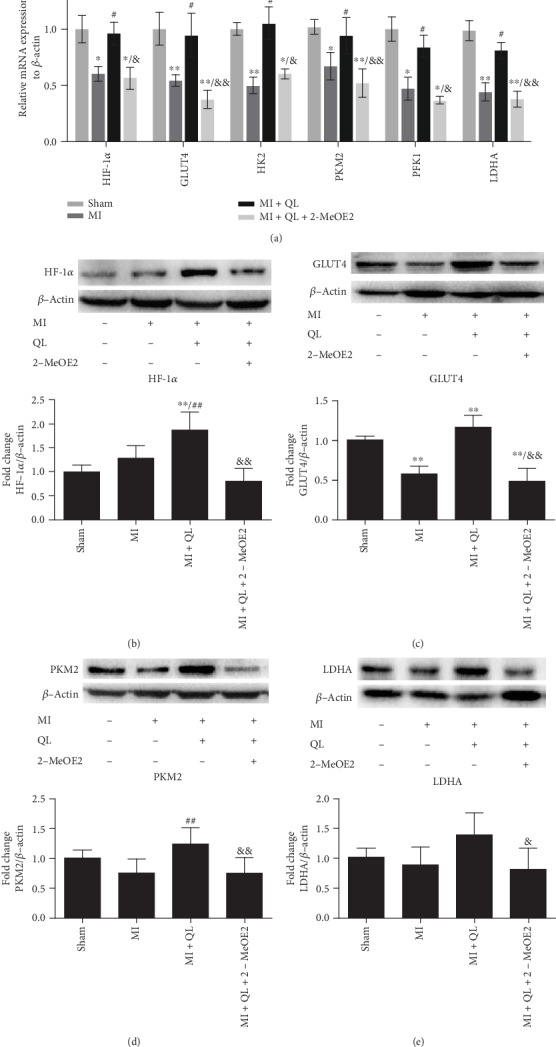
QL treatment promoted myocardial glucose uptake and glycolysis in a HIF-1*α*-dependent manner. (a) QL upregulated mRNA expression of HIF-1*α*, GLUT4, HK2, PKM2, PFK1, and LDHA, which were significantly abolished by 2-MeOE2 (*n* = 7/group). (b–e) QL upregulated protein expressions of HIF-1*α*, GLUT4, PKM2, and LDHA, which were also abolished by 2-MeOE2 (*n* = 7/group). Data are expressed as means ± SEM. ^∗^*p* < 0.05 and ^∗∗^*p* < 0.01 compared with the sham group; ^#^*p* < 0.05 and ^##^*p* < 0.01 compared with the MI group; ^&^*p* < 0.05 and ^&&^*p* < 0.01 compared with the MI+QL group.

Moreover, both mRNA and/or protein expressions of HIF-1*α*, GLUT4, HK2, PKM2, PFK1, and LDHA were upregulated in the MI+QL group compared with MI group, while the HIF-1*α* inhibitor attenuated these effects (Figures 4(a)–4(e)). HK2, PKM2, PFK1, and LDHA are the key enzymes of glycolysis, and GLUT4 was the key glucose transporter, so these results further indicated that QL promoted myocardial glucose uptake and glycolysis via the HIF-1*α* signaling pathway.

### 3.4. QL Treatment Activated Enzyme Expression of Glucose Oxidation and Serum Fatty Acid Uptake in a HIF-1*α*-Independent Manner

**Figure 5:**
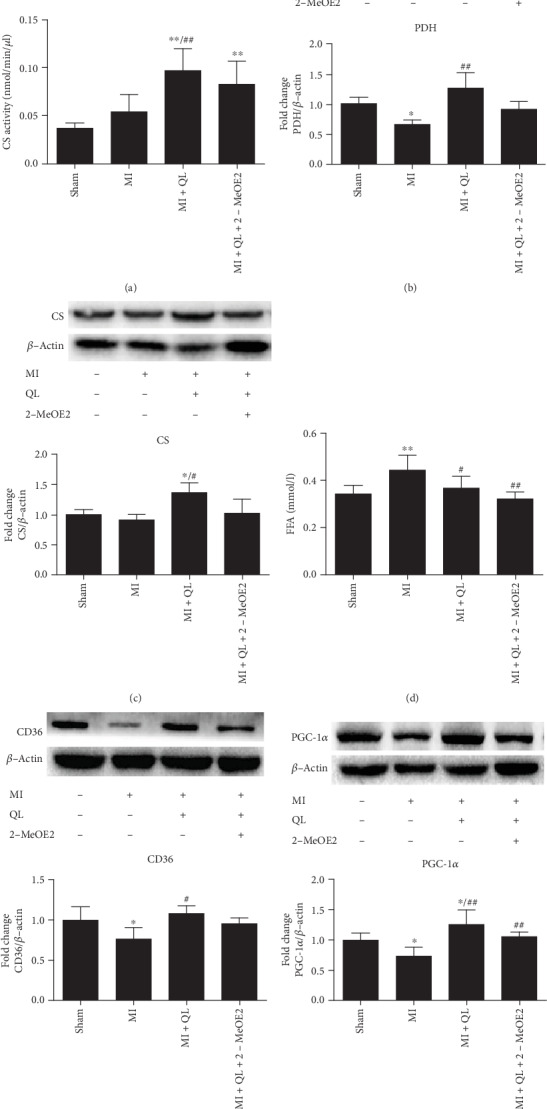
QL treatment promoted glucose oxidation and free fatty acid (FFA) uptake in a HIF-1*α*-independent manner. (a) QL promoted CS protein activity (*n* = 7/group). (b and c) QL elevated PDH and CS protein expressions, while the HIF-1*α* inhibitor did not significantly attenuate this effect (*n* = 7/group). (d) QL decreased serum FFA levels (*n* = 7/group). (e and f) QL significantly promoted FFA uptake by increasing the expressions of CD36 and PGC-1*α*, which were downregulated in the MI group, and the HIF-1*α* inhibitor could not reversed this effect (*n* = 7/group). Data are expressed as means ± SEM. ^∗^*p* < 0.05 and ^∗∗^*p* < 0.01 compared with the sham group; ^#^*p* < 0.05 and ^##^*p* < 0.01 compared with the MI group.

PDH and CS are key enzymes involved in glucose aerobic oxidation. We found that protein expressions of PDH and CS, in addition to CS activity, were significantly increased after QL, while HIF-1*α* inhibition did not significantly affect this effect (Figures 5(a)–5(c)). The present study also demonstrated significantly decreased CD36 and PGC-1*α* protein expressions and increased serum FFA levels in the MI group compared with the sham group. QL treatment upregulated CD36 and PGC-1*α* expressions and lowered serum FFA levels, which was not significantly affected by HIF-1*α* inhibition (Figures 5(d)–5(f)**)**. This indicates that QL promoted fatty acid uptake in a HIF-1*α*-independent manner.

### 3.5. QL Treatment Promoted Microvascular Angiogenesis of Myocardial Tissue via a HIF-1*α*/VEGF Pathway

**Figure 6:**
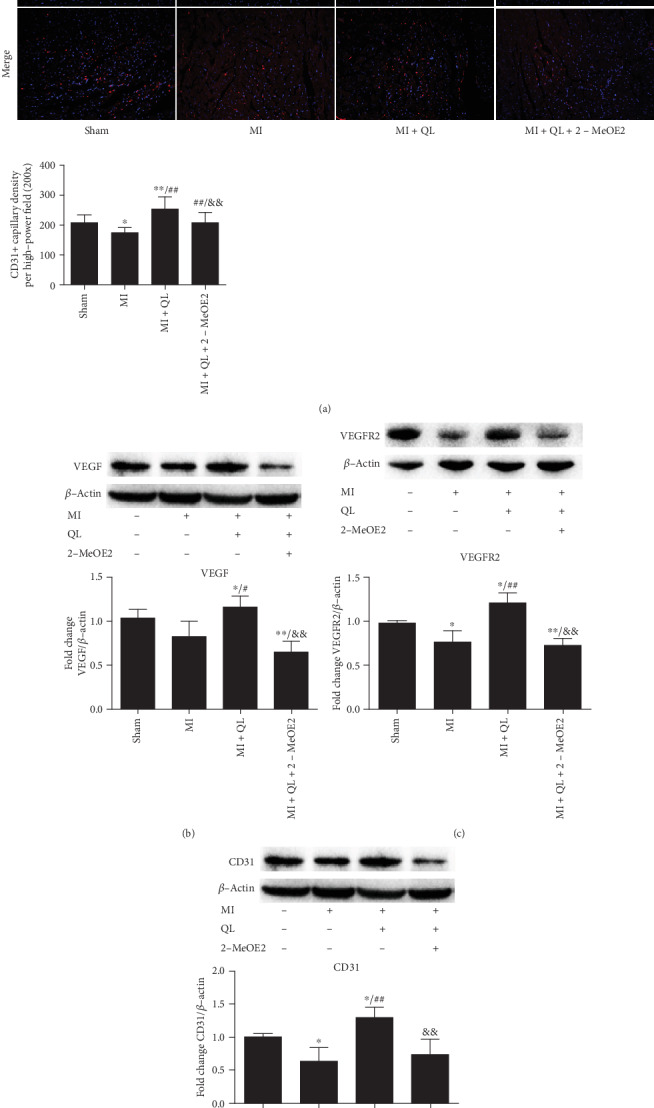
QL treatment promoted myocardial angiogenesis. (a) CD31 immunofluorescence staining (20x, scale bar = 100 *μ*m) showed that QL significantly increased myocardial angiogenesis, which was significantly attenuated by pretreatment with the HIF-1*α* inhibitor. Red indicates CD31-positive signals; blue indicates DAPI nuclei. The newborn capillary density was quantified as the number per high-power field (*n* = 4/group). Western blot analysis showed that QL significantly upregulated myocardial VEGF (b), VEGFR2 (c), and CD31 (d) protein expressions, while pretreatment with the HIF-1*α* inhibitor attenuated these effects (*n* = 7/group). Data from at least four independent experiments were calculated and expressed as means ± SD. ^∗^*p* < 0.05 and ^∗∗^*p* < 0.01 compared with the sham group; ^#^*p* < 0.05 and ^##^*p* < 0.01 compared with the MI group; ^&&^*p* < 0.01 compared with the MI+QL group.

CD31 immunofluorescence staining and Western blot analysis were performed to detect the effects of QL on myocardial microvascular density. Representative images are shown in Figure 6(a). CD31-positive endothelial cells are shown in red and nuclei in blue. Our data revealed a significantly higher CD31-positive microvascular density in the MI+QL group than in the MI group, which was significantly attenuated by treatment with 2-MeOE2. Western blot results also showed that QL could upregulate VEGF, VEGFR2, and CD31 expression, while treatment with a HIF-1*α* inhibitor attenuated the QL-induced effect (Figures 6(b)–6(d)). Collectively, our data implied that QL treatment promoted the angiogenesis of infarcted myocardium partly via upregulating the HIF-1*α*/VEGF pathway.

## 4. Discussion

In the present study, we observed the effects of QL on myocardial energy metabolism and explored the underlying mechanisms. The results revealed that QL reversed myocardial remodeling by promoting glycolysis via the HIF-1*α*-dependent pathway, while QL can also increase the enzyme expression of glucose oxidation and free fatty acid uptake via a HIF-1*α*-independent mechanism.

The reduced substrate utilization capacity served as one important pathological mechanism of cardiac dysfunction in heart failure, which was the result of reduced substrate uptake, oxidation, or a combination of both [25]. There are conflicting results in the previous studies regarding substrate utilization in heart failure [26]. These previous studies found that glucose utilization was increased in early heart failure but substantially declined in advanced heart failure [27–30]. Results on the changes in fatty acid utilization in heart failure are also inconsistent [26], but most studies show that it remained unchanged or slightly increased early in heart failure, before having decreased in advanced heart failure [31–33]. In this study, we found that compared with the sham group, myocardial glucose uptake, oxidation, and ATP production were reduced, while the serum FFA level was elevated in MI rats. These factors might contribute to the observed cardiac dysfunction in MI rats. QL treatment significantly increased myocardial glucose uptake, glycolysis, oxidation, and ATP production; decreased serum FFA; and improved cardiac function. The upregulation of the levels of HIF-1*α*, GLUT4, PDH, CD36, and PGC-1*α* expression was involved in the observed beneficial effects of QL.

HIF-1*α* is a master transcription factor that regulates many target genes (such as VEGF, VEGF receptor 1, and GLUT4), whose protein products play a critical role in improving angiogenesis and vascular remodeling, glucose metabolism, cell survival, and oxygen delivery [12, 34–38]. VEGF is a major mediator of neovascularization in physiological and pathological conditions by promoting vessel formation [39]. Significantly upregulated HIF-1*α* and VEGF have been found in the rat ischemic myocardium 1 week after MI, while their expressions were only slightly increased compared with the sham group 2 weeks and 4 weeks after MI [40–42]. The expression levels of VEGF and HIF-1*α* were positively related with the increasing microvessel density in the infarct area. In the present study, the protein expressions of HIF-1*α* and VEGF were similar between the two groups. The reason may be that the initial activation of HIF-1*α* and VEGF promotes angiogenesis and improves blood and oxygen supply, which leads to the gradual decrease of expression. QL treatment significantly upregulated their expressions and promoted neovascularization. Results from the CD31 immunofluorescence staining and Western blot assay also demonstrated that QL increased neonatal capillary density and CD31 protein expressions, while inhibition of HIF-1*α* and VEGF activity abolished these beneficial effects of QL.

In addition to promoting angiogenesis and oxygen delivery, HIF-1*α* also activates several downstream genes encoding transporters and enzymes related to glucose metabolism. Glucose transportation into cardiomyocytes determines the efficiency of glucose utilization. This is insulin dependent and requires the most important (quantitatively) glucose transporter expressed in the myocardium, which can be activated by HIF-1*α*. Previous studies have shown that the myocardial expression of GLUT4 is reduced in rats with heart failure [18, 43], which leads to impaired cardiac glucose metabolism. Metabolic gene expression in the failing human heart showed decreased GLUT1, GLUT4, and PDK2 mRNA expression [29]. Consistent with previous studies, we found that the mRNA and protein expressions of GLUT4 in the myocardium of MI rats were significantly reduced. Importantly, compared with the MI group, QL treatment significantly upregulated GLUT4 mRNA and protein expressions, which were attenuated by the HIF-1*α* inhibitor. Furthermore, PET imaging also suggested that QL could promote myocardial glucose uptake by regulating the HIF-1*α* signal pathway.

Apart from glucose transporters, several key enzymes including HK2 [36, 44], PFK1, and PKM2 [45] play an important role in glucose metabolism by converting glucose to pyruvate, which is also regulated by HIF-1*α*. Pyruvate can be converted to acetyl-CoA by PDH or to lactic acid by LDHA. HIF-1*α* can activate pyruvate dehydrogenase kinase 1 (PDK1) and LDHA expression to switch cells from aerobic metabolic to anaerobic glycolysis [46, 47], since PDH can be phosphorylated and inactivated by PDK1. In our study, we found that compared with the sham group, the mRNA expressions of HK2, PFK1, PKM2, and LDHA were reduced, but the protein expression of PKM2 and LDHA remained unchanged in the MI group; QL treatment significantly upregulated their expressions, which could be suppressed by pretreatment with the HIF-1*α* inhibitor. These results indicated that QL could promote the myocardial glycolysis of post-MI hearts in a HIF-1*α*-dependent manner.

Moreover, QL also increased PDH and CS activity in a HIF-1*α*-independent manner. CS is a mitochondrial enzyme found in all cells that is capable of oxidative metabolism and is involved in lipogenesis, cholesterogenesis, and energy production. Its activity follows a circadian pattern, with the results indicating that QL could also enhance glucose aerobic metabolism. However, HIF-1*α*-mediated induction of PDK1 has been found to prevent the entry of pyruvate into the tricarboxylic acid cycle [46], which seems contradictory to our results. Nevertheless, activated HIF-1*α* could promote angiogenesis as well as recover blood and oxygen supply, which might favor mitochondrial respiration and aerobic metabolism. Thus, this may explain these conflicting outcomes. On the other hand, our results showed that QL promoted PDH and CS expression via HIF-1*α*-independent pathways.

PGC-1*α* is a powerful transcriptional coactivator and a key regulator in cardiac mitochondrial biogenesis and oxidative metabolism, which works by cooperating with numerous transcription factors (such as nuclear respiratory factor-1 or 2 and estrogen-related receptor *α*) to promote related genes [48]. It plays a critical role in upregulating antioxidant genes and interacting with peroxisome proliferator-activated receptors (PPARs) to increase fatty acid oxidation and angiogenesis [49]. Fatty acid translocase (FAT)/CD36 is a key protein in the translocation of fatty acids across the sarcolemmal membrane of cardiomyocytes [5]. CD36 and CPT-1 are the target genes of PPAR, which was coactivated by PGC-1*α*. There were studies showing that QL could increase PGC-1*α* and CPT-1 expression in rats with heart failure from pressure overload and H9C2 cardiomyocytes [18, 19]. Due to heart failure, serum FFA concentrations increased significantly, which could decrease glucose oxidation by inhibiting PDH activity [7]. On the other hand, there were studies presenting decreased circulating FFA concentrations, which could promote glucose uptake and oxidation indirectly [9]. In accordance with a previous study, we found that QL significantly upregulated the expression of the PGC-1*α* and CD36 protein, which may promote FFA uptake and decrease serum FFA levels in a HIF-1*α*-independent pathway. It is important to note that decreased serum FFA concentration might further promote glucose oxidation indirectly through activating PDH expression. A previous study also showed that QL and its main chemical property (astragaloside) could improve diastolic and systolic function by promoting AMPK phosphorylation and PPAR*γ*, PPAR*α*, and PGC-1*α* expression [18, 50–54], which are key regulators of energy metabolism. So, we postulate that the AMPK, PPAR, and PGC-1*α* pathway might be the possible mechanisms for QL to promote aerobic metabolism, and which were independent of the HIF-1*α* pathway.

## 5. Conclusions

In summary, the present study demonstrated that QL restored the microstructure of myocardium, attenuated myocardial fibrosis, and improved cardiac function in heart failure rats post MI. In the present study, we found that the enhancement of enzyme expression of myocardial glucose and fatty acid metabolism with elevated ATP level may account for the observed beneficial effects of QL. The activation of the HIF-1*α* signaling pathway and its downstream target genes proved to be at least partly involved in these benefits, including promoted glycolysis and ATP level. Although the effect of QL on promoting enzyme expression of glucose oxidation and FFA uptake is achieved via a HIF-1*α*-independent pathway, the exact mechanism behind this effect requires further study. Our data provided a novel potential working mechanism of QL regarding its impact on myocardial glucose and fatty acid metabolic balance with an elevated ATP level in this rat model of heart failure after myocardial infarction.

## Figures and Tables

**Table 1:** Primer sequences for HIF-1*α* and glucose metabolism-related genes.

Genes	Forward primer	Reverse primer
HIF-1*α*	5′-CTCCCTTTTTCAAGCAGCAG-3′	5′-GCTCCATTCCATCCTGTTCA-3′
GLUT4	5′-AGGCACCCTCACTACCCTTT-3′	5′-AGCATAGCCCTTTTCCTTCC-3′
HK2	5′-CTCTGGGTTTCACCTTCTCG-3′	5′-ACCACATCTCTGCCTTCCAC-3′
PFK1	5′-GTTCTGGGGATGCGTAAGAG-3′	5′-CCTCAGTTTCAGCCACCACT-3′
PKM2	5′-TCCCATTCTCTACCGACCTG-3′	5′-TTCAGTGTGGCTCCCTTCTT-3′
LDHA	5′-GTCAGCAAGAGGGAGAGAGC-3′	5′-CACTGGGTTTGAGACGATGA-3′
*β*-Actin	5′-GAAGTGTGACGTTGACATCCG-3′	5′-TGCTGATCCACATCTGCTGGA-3′

## Data Availability

The data used to support the findings of this study are available from the corresponding author upon request.
